# Remote Stab Wound Resulting in AV Fistula and High-Output Heart Failure

**DOI:** 10.1155/2013/902719

**Published:** 2013-08-05

**Authors:** Jennifer A. Rymer, Lindsay L. Anderson, J. Trevor Posenau, W. Schuyler Jones

**Affiliations:** Duke University Medical Center, Hospital North 7402, DUMC Box 3126, Durham, NC 27710, USA

## Abstract

A 54-year-old African American male with no medical history presented to an urgent care clinic with signs and symptoms of new-onset congestive heart failure. There was an initial concern for congestive heart failure secondary to an ischemic etiology as an echocardiogram revealed a depressed ejection fraction. However, a left heart cardiac catheterization did not demonstrate any significant coronary disease. As a loud bruit was auscultated over the right base of the patient's neck, he underwent a carotid duplex ultrasound revealing a fistula between the right common carotid artery (CCA) and the right internal jugular vein (IJV). A diagnosis of high-output heart failure secondary to a large arteriovenous (AV) fistula was made, and the patient underwent ligation and repair of the fistula with resolution of symptoms of congestive heart failure.

## 1. Introduction

The primary etiologies of congestive heart failure, particularly in older adult patients, are hypertension and coronary artery disease [1]. A rare cause of congestive heart failure includes systemic AV shunting or vasodilation, eventually over time resulting in a high-output heart failure state [2]. Large AV fistulas formed after lumbar disc surgery, cardiac catheterization, and for hemodialysis access have been increasingly recognized as iatrogenic causes of high-output heart failure [3–5]. However, few cases of remote trauma resulting in a high-output heart failure state are documented in the literature.

## 2. Case Report

A 54-year-old African American male with no past medical history presented to the urgent care clinic with two weeks of bilateral lower extremity edema and dyspnea on exertion. Physical exam revealed a palpable thrill over the right base of the neck with a loud continuous bruit. A healed wound was prominent and had been present since he was stabbed in the neck by his girlfriend thirty years ago. Additionally, there was elevated jugular venous distention to the earlobe at 30 degrees, and the point of maximum impulse was displaced inferolaterally on cardiac exam. S1 and S2 were irregularly irregular with no additional heart sounds. Lungs were clear to auscultation bilaterally; however, the patient had 2+ edema extending up to his knees bilaterally.

A transthoracic echocardiogram on admission revealed an ejection fraction of 30%, an estimated right ventricular systolic pressure of 49 mm Hg, and a severely dilated right ventricle. Left heart cardiac catheterization revealed no significant coronary artery disease. However, right heart catheterization was significant for cardiac output of 12.8 L/min, cardiac index of 6.6 L/min/m^2^, right atrial pressure of 17 mm Hg, and right ventricular pressure of 50/15. A 4 : 1 left-to-right shunt was calculated using the Flamm equation.

**Figure 1:**
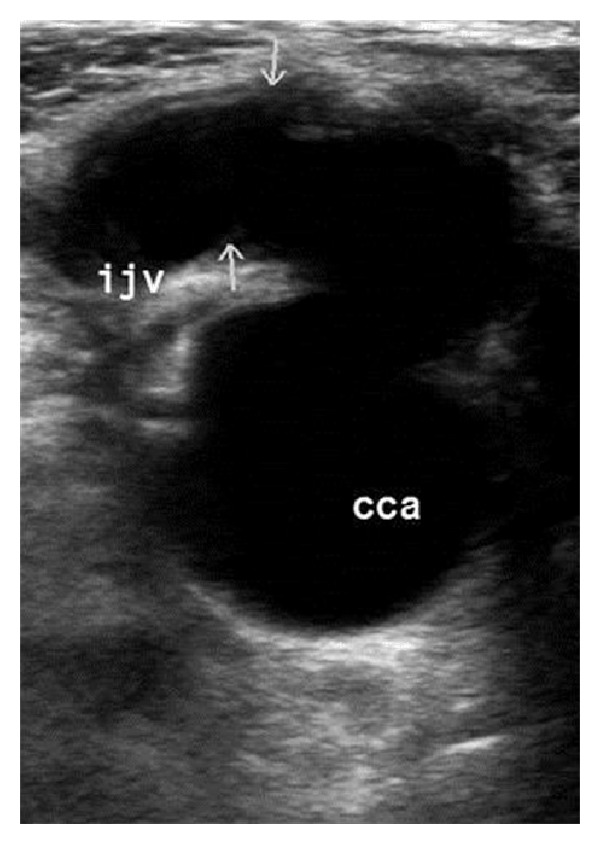
Ultrasound demonstrating fistula connecting right CCA to the right IJV.

Carotid duplex ultrasound (Figure 1) demonstrated a large pseudoaneurysm arising from the right CCA with a communication to the right IJV. During a combined vascular and thoracic surgery operation, a 0.7 cm fistula was ligated between the right CCA and the right IJV. The right CCA was repaired with a Dacron patch. With the exception of perioperative atrial fibrillation with rapid ventricular response (treated with amiodarone, diltiazem infusion, and successful DC cardioversion), the patient's symptoms continued to improve prior to discharge.

## 3. Discussion

There is sparse literature documenting traumatic cases of large AV fistulas leading to high-output heart failure. High-output congestive heart failure is often associated with severe anemia, hyperthyroidism, various vitamin deficiencies, and rarely large AV fistulas [2]. These AV fistulas can be congenital, traumatic, or iatrogenic and result in decreased systemic vascular resistance [2, 6]. Iatrogenic causes include AV fistulas resulting from lumbar disc surgery and cardiac catheterization [4, 5].

The two mechanisms resulting in high-output heart failure over a course of months to years are vasodilation and AV shunting [2]. Disease states, such as severe chronic anemia [7] and sepsis [8], lead to systemic vasodilation. Conditions including McCune-Albright [2], large AV fistulas, and multiple myeloma [9] produce systemic AV shunting. Sympathetic activation and decreased renal perfusion contribute to ventricular remodeling and clinical heart failure [2]. Although the most common cause of new-onset heart failure is ischemic heart disease, this case documents the value of a thorough history and physical exam in detecting less common, and potentially treatable, causes [10]. This case highlights the importance of the identification and treatment of acquired AV fistulas in the pathophysiology of high-output heart failure.
